# Les corps étrangers en ORL: expérience de dix ans

**DOI:** 10.11604/pamj.2015.21.91.6975

**Published:** 2015-06-05

**Authors:** Khaoula Hssaine, Btissam Belhoucha, Youssef Rochdi, Hassan Nouri, Lahcen Aderdour, Abdelaziz Raji

**Affiliations:** 1 Service d'ORL et de Chirurgie Cervico-faciale, CHU Mohammed VI, Marrakech, Maroc

**Keywords:** Corps étranger, voies aéro-digestive supérieure, oreille, enfants, adultes, foreign body, upper aero-digestive tract, ears, child, adult

## Abstract

Les corps étrangers (CE) représentent une pathologie fréquemment rencontrée en pratique ORL d'urgence. Ils peuvent constituer une urgence vitale par leur aspect ou leur siège. Nous présentons le bilan de dix ans sur la prise en charge des CE de la sphère ORL avec une revue de la littérature. Il s'agit d'une étude rétrospective entre Janvier 2004 et Décembre 2013, incluant tous les cas de CE des voies aéro-digestives supérieures et auriculaires colligés dans le service d'ORL au CHU Mohammed VI de Marrakech au Maroc. Sur les 1317 cas de CE de la sphère ORL colligés durant cette période, 80,48% concernaient les enfants. Le sex-ratio était de 1,5. L’âge moyen était de 12,92 ans. Les CE œsophagiens étaient les plus fréquents (47,53%). Les complications ont été rencontrées dans 11,69% des cas. Les CE dans la sphère ORL restent fréquents en pratique quotidienne surtout chez les enfants. Leur prise en charge nécessite une intervention rapide avec un matériel adapté et des médecins entrainés. La prévention reste la meilleure solution.

## Introduction

Les corps étrangers sont des urgences fréquentes en ORL. Ils peuvent engager parfois le pronostic vital par leur siège ou leur nature. Les publications concernant ce sujet sont peu fréquentes et portent surtout sur des périodes limitées. L'objectif de l’étude est d'exposer notre expérience concernant la prise en charge des corps étrangers au niveau de la sphère ORL pendant dix ans, avec les données actuelles sur ce sujet.

## Méthodes

Il s'agit d'une étude rétrospective entre janvier 2004 et décembre 2013 incluant tous les cas de corps étranger des VADS et du conduit auditif externe colligés au service d'ORL et de chirurgie cervico-faciale au CHU Mohammed VI de Marrakech au Maroc. Les données étudiées comportaient: l’âge, le sexe, le motif et le délai de consultation, la localisation, la nature des corps étrangers, le diagnostic, l'extraction et l’évolution. Les corps étrangers trachéo-bronchiques et les patients dont l'exploration était blanche ont été exclus.

## Résultats

Epidémiologie: nous avons recensé 1317 cas de corps étrangers de la sphère ORL durant cette période. Ce motif de consultation représentait 7,9% de l'ensemble des urgences ORL dans notre service. Mille soixante cas concernaient les enfants (soit 80,48%). Le sex-ratio était de 1,5. L’âge des patients était compris entre 18 mois et 80 ans. L’âge moyen était de 12,92 ans, avec un pic de fréquence chez les enfants âgés de moins de 5 ans.

La localisation: les CE ont été retrouvés dans l’œsophage (47,53%), les fosses nasales (25,81%), l'oreille (16,24%), le pharynx (8,27%), et le larynx (2,12%). Cet ordre était applicable au groupe pédiatrique (moins de 15 ans), par contre les CE dans le conduit auditif externe prédominaient chez l'adulte.

La durée de séjour du CE: la durée moyenne de séjour du CE dans la sphère ORL était de 5,34 jours (1heure-4 ans). Cinq cent vingt patients soit 39,48% consultaient après 24 heurs de l'incident. Cent soixante quinze CE soit 13,28% des corps étrangers ont été déjà manipulés surtout les CE nasaux et auriculaires.

**Figure 1:**
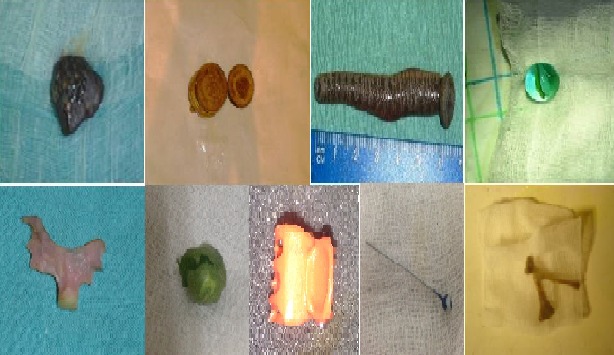
Exemples de corps étrangers de la sphère ORL après extraction

La nature des CE: les corps étrangers inertes représentaient la majorité des CE de la sphère ORL (97,41%) (Figure 1). Le Tableau 1 représente la nature des CE retrouvés en fonction du siège au niveau de la sphère ORL.

**Tableau 1:** La nature des corps étrangers en fonction du siège au niveau de la sphère ORL

Siège Nature	Œsophage (nombre)	Fosse nasale (nombre)	Oreille (nombre)	Pharynx (nombre)	Larynx (nombre)
**Pièce de monnaie**	511	-	-	2	-
**Morceau d'os**	42	-		30	5
**Arrête de poisson**	26	-	-	36	2
**Viande**	4	-	-	-	-
**Plastique**	14	21	9	6	4
**Métallique**	6	7	2	7	2
**Dentier**	9	-	-	2	2
**Pile bouton**	6	3	3	1	-
**Sangsue**	1	2	-	18	7
**Grains**	-	141	30	-	-
**Coton**	-		85	-	-
**Papier**	-	44	-	-	-
**Eponge**	-	27	-	-	-
**Pierre**	-	24	4	-	-
**Ail**	-	-	36	-	-
**Perle**	-	50	34	-	-
**Insecte**	-	-	6	-	-
**Cacahuète**	-	-		-	3
**Autres ou non reconnus: craie, crayon, gomme, Chwingum …**	7	21	5	7	3

**Figure 2:**
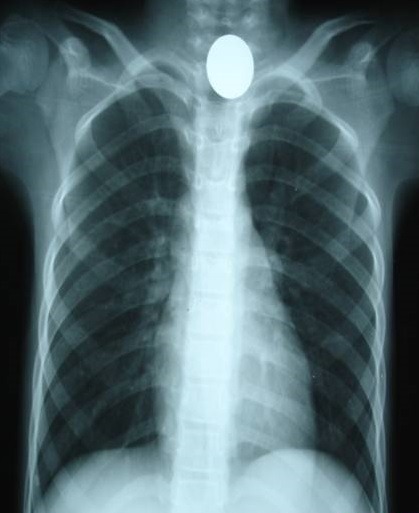
Radiographie thoracique objectivant un corps étranger radio-opaque œsophagien (pièce de monnaie)

Le diagnostic: la symptomatologie clinique était très variable. Le Tableau 2 résume les modes de révélation en fonction du siège du CE. Les radiographies standards étaient réalisées de façon systématique pour les localisations pharyngées et œsophagienne; pour localiser les corps étrangers métalliques et osseux (Figure 2).

**Tableau 2:** La symptomatologie révélatrice du corps étranger en fonction du siège

Siège	Symptômes	Nombre	Pourcentage
**Œsophage et pharynx**	Odynophagie	706	96,05
	Dysphagie	567	77,14
	Hypersialorrhée	415	56,46
	Fièvre	1	0,13
	Sensation de CE	187	25,44
	Amaigrissement	1	0,13
	Dyspnée	1	0,13
	hématémèse	5	0,68
	épistaxis	4	0,54
	Anémie	5	0,68
**Fosses nasales**	Rhinorrhées purulente unilatérale	208	61,17
	Cacosmie	128	37,64
	Obstruction nasale	200	58,82
	Rhinorrhée purulente bilatérale	4	1,17
	Epistaxis	7	2,05
	fièvre	3	0,88
	Rhinolithiase	1	0,29
	Anémie	2	0,58
	Aucun symptôme	105	30,88
**Oreille**	Otalgie	97	45,32
	Hypoacousie	8	3,73
	Otorragie	9	4,20
	Otorrhées purulentes	13	6,07
	Acouphènes	24	11,214
	Aucun symptôme	73	34,11
**Larynx**	dyspnée	11	39,28
	dysphonie	3	
	Syndrome de pénétration	21	75
	Sensation de CE	7	25
	Hématémèse	5	17,85
	Asphyxie	1	3,57

Prise en charge thérapeutique: l'extraction des CE a été faite le plus souvent par les voies naturelles, réalisée avec ou sans anesthésie locale en salle de consultation des urgences. Le recours à l'anesthésie générale au bloc opératoire était nécessaire dans 54,89% des cas. La chirurgie par voies externe était réalisée chez 3 patients pour un CE œsophagien négligé compliqué d'une sténose œsophagienne et deux cas de CE du conduit auditif externe migrant dans l'oreille moyenne à travers une perforation tympanique (Tableau 3). Un traitement antibiotique local ou général a été prescrit à visée curative dans les complications infectieuses; et à visée prophylactique devant les lésions muqueuses. Une restriction provisoire de l'alimentation orale a été préconisée devant les lésions muqueuses œsophagiennes. Le traitement martial a été prescrit chez les 7 cas d'anémie. L’éducation des parents et des patients pour prévenir un deuxième incident était systématique.

**Tableau 3:** Les modalités d'extraction du corps étranger en fonction du siège dans la sphère ORL

Siège	Modalités d'extraction	Nombre
**Œsophage cervicale**	Endoscopie rigide sous anesthésie générale	625
	Chirurgie par voie externe	1
**Fosses nasales**	Extraction à la pince ou crochet	305
	Aspiration	25
	Association des 2 méthodes précédentes	6
	Endoscopie sous anesthésie générale	4
**Oreille**	Extraction par micro-instruments sous microscope	139
	Aspiration	34
	Association des 2 méthodes précédentes	20
	Extraction sous microscope avec anesthésie générale	19
	Chirurgie par voie externe	2
**pharynx**	Extraction à la pince	65
	Endoscopie rigide sous anesthésie générale	44
**Larynx**	Endoscopie rigide sous anesthésie générale	27
	Trachéotomie première	1

Evolution: l’évolution était souvent favorable après extraction du CE. Les complications représentaient 11,69%; survenant dans 53,89% des cas chez des patients dont l'extraction a été déjà tentée avant la consultation. Les complications les plus fréquentes étaient les lésions du CAE et de la muqueuse digestive suivie par les complications infectieuses. La mortalité était de 0,22% suite à deux cas de médiastinite secondaire à une pile bouton et un morceau d'os de localisation œsophagienne ancienne, et un cas d'asphyxie secondaire à un CE laryngé enclavé.

## Discussion

Les corps étrangers (CE) représentent une pathologie fréquemment rencontrée en pratique ORL d'urgence. Ils représentent selon les auteurs en moyenne 11% de l'ensemble des urgences ORL. Ils peuvent survenir à tout âge à partir de l’âge de préhension et surtout chez l'enfant de moins de 6 ans, avec une nette prédominance masculine [1, 2]. Le CE est généralement accidentel survenant lors du jeu ou des repas. Il survient habituellement chez des personnes ayant un développement normal. Il peut être favorisé par un terrain particulier: retard mental, trisomie ou toute autre infirmité psychomotrice. Dans la majorité des publications [2–5], la localisation auriculaire prédomine entre 44% et 68%. Dans notre étude, nous avons constaté que les CE œsophagiens représentent l'incidence la plus élevée (47,53%). Ce résultat était similaire à l’étude menée par Kitcher E et al [6]. La nature du CE varie en fonction de l’âge, la localisation, et les particularités sociodémographiques [7]. Les CE inertes d'origine alimentaire prédominent dans la littérature [5]. Les CE vivantes sont peu fréquents (2,58% dans notre série), mais ils sont responsable de plus de complications corrélées à la durée de séjour dans la sphère ORL. Les pièces de monnaies sont les CE les plus retrouvés au niveau de l’œsophage chez l'enfant [5, 7]. Les piles boutons constituent un cas à part, particulièrement dangereux. Leur extraction est une extrême urgence avant l'apparition des complications [8]. La durée de séjour d'un corps étranger est éminemment variable de quelques minutes à plusieurs mois. Le diagnostic est évident lors d'un accident survenant en présence de l'entourage ou rapporté par le patient lui-même, mais peut parfois être méconnu surtout chez le jeune enfant. La symptomatologie clinique est non spécifique et variable en fonction du siège. Les examens complémentaires sont rarement nécessaires pour le diagnostic, puisque la majorité des CE sont radio-transparents [9]. La visualisation directe lors de l'examen clinique et endoscopique à l'optique rigide ou au nasofibroscope sont généralement suffisants pour identifier et localiser les CE.

La localisation laryngée est la plus redoutée des CE en pratique ORL car elle est source de morbidité et de mortalité en particulier chez l'enfant de moins de 3 ans. Ce diagnostic doit être évoqué devant toute détresse respiratoire aigue de l'enfant et nécessite une prise en charge en extrême urgence. Le traitement consiste à l'extraction la plus atraumatique possible du CE. Plusieurs techniques sont décrites, et le choix dépend de la localisation, le type du CE, l’âge du patient et l'expérience du médecin [10–12]. L'extraction se fait souvent sans ou sous anesthésie locale par les voies naturelles, sous guidage visuel sous miroir de Clar, à l'aide du microscope ou de l'endoscopie rigide ou souple. L'usage de pinces spécifiques pour l'extraction est la technique la plus utilisée. L'exploration ORL œsophagienne et pharyngée blanche chez un patient symptomatique doit impérativement amener à une exploration endoscopique par des gastro-entérologues. Le traitement chirurgical par voie externe est devenu rare et ne concerne que les CE anciens et difficiles à extraire par les méthodes usuelles, ou en cas de complications (perforation ou sténose) [9]. Le recours à l'anesthésie générale pour l'extraction des CE dans la littérature varie entre 8,6% et 30% des cas. Il concerne les CE œsophagiens et laryngé, les patients non coopérant et d’âge jeune, ou après échec de plusieurs tentatives d'extraction [3]. Le taux élevé d'usage d'anesthésie générale dans notre série (54,89% des cas) peut être expliqué par le l'incidence élevée des CE œsophagiens et la fréquence des enfants très jeune qui ne tolèrent pas souvent l'anesthésie locale. Les complications des CE de la sphère ORL sont le plus souvent simples, mais peuvent être graves comme l'obstruction des voies respiratoires supérieures, les perforations viscérales et les infections graves (médiastinite, pneumopathie) [13]. Le taux des complications peut atteindre jusqu’à 22%. La plupart des auteurs rapportent une incidence plus élevée de complications iatrogènes en cas de manipulation précédente [2, 3]. Les complications dépendent aussi d'autres facteurs: la coopération du patient, l'expérience du médecin, le type du CE, la durée de séjour, la visibilité et la profondeur du CE et la disponibilité d'un équipement adapté. La sensibilisation des personnels de la santé pour référer les patients présentant des CE aux urgences ORL dans les plus brefs délais permettra de réduire le taux des complications. Ainsi la surveillance des enfants au moment des jeux et des repas restera la meilleure solution pour réduire l'incidence des CE.

## Conclusion

Les corps étrangers de la sphère ORL restent un motif fréquent en pratique ORL d'urgence; surtout chez l'enfant après l’âge de préhension. Leur diagnostic est souvent facile, mais peuvent parfois être fatales par leur siège ou leur nature. Dans notre étude les CE œsophagiens prédominent chez l'enfant et la localisation auriculaire prédomine chez l'adulte. Les pièces de monnaies, les grains et le coton sont les CE les plus fréquents dons notre contexte. La majorité des complications sont observées chez les patients dont l'extraction a été déjà tentée de façon inadaptée. La prise en charge des CE nécessite un matériel adapté et des médecins ORL entrainés. La prévention reste la meilleure solution et passe par la sensibilisation des parents, des patients et des personnels de santé.
